# Preventing Compulsory Admission to Psychiatric Inpatient Care: Perceived Coercion, Empowerment, and Self-Reported Mental Health Functioning after 12 Months of Preventive Monitoring

**DOI:** 10.3389/fpsyt.2015.00161

**Published:** 2015-11-18

**Authors:** Barbara Lay, Thekla Drack, Marco Bleiker, Silke Lengler, Christina Blank, Wulf Rössler

**Affiliations:** ^1^Department for Psychiatry, Psychotherapy and Psychosomatics, University Hospital of Psychiatry Zurich, Zurich, Switzerland; ^2^The Zurich Program for Sustainable Development of Mental Health Services (ZInEP), University Hospital of Psychiatry Zurich, Zurich, Switzerland; ^3^Laboratory of Neuroscience (LIM27), Institute of Psychiatry, University of São Paulo, São Paulo, Brazil

**Keywords:** perceived coercion, empowerment, compulsory hospitalization, prevention, evaluation

## Abstract

**Objective:**

To evaluate the effects of a preventive monitoring program targeted to reduce compulsory rehospitalization and perceived coercion in patients with severe mental disorder. We analyze patient outcomes in terms of perceived coercion, empowerment, and self-reported mental health functioning at 12 months.

**Methods:**

The program consists of individualized psychoeducation, crisis cards and, after discharge from the psychiatric hospital, a 24-month preventive monitoring. In total, 238 psychiatric inpatients who had had compulsory admission(s) during the past 24 months were included in the trial. T1-assessment 12 months after baseline was achieved for 182 patients.

**Results:**

Study participants reported lower levels of perceived coercion, negative pressures, and process exclusion, a higher level of optimism, and a lesser degree of distress due to symptoms, interpersonal relations, and social role functioning (significant time effects). However, improvements were not confined to the intervention group, but seen also in the treatment-as-usual group (no significant group or interaction effects). Altered perceptions were linked to older age, shorter illness duration, female sex, non-psychotic disorder, and compulsory hospitalization not due to risk of harm to others.

**Conclusion:**

Our findings suggest that changes in the subjective perspective were fueled primarily by participation in this study rather than by having received the specific intervention. The study contributes to a better understanding of the interaction between “objective” measures (compulsory readmissions) and patients’ perceptions and highlights the need for treatment approaches promoting empowerment in individuals with a history of involuntary psychiatric hospitalizations.

## Introduction

Compulsory admission to psychiatric hospital treatment constitutes a serious intervention that affects an individual’s personal interests and autonomy profoundly. Notwithstanding that such measures are essential to preventing persons at risk of endangering themselves or others from doing harm, if these persons cannot be helped by other means in a less restrictive setting, patients may perceive such measures as being unjustified or harmful (1). Moreover, they may have adverse effects on the therapist–patient relationship and be associated with negative outcomes (2–5). In some countries, including Switzerland, comparatively high rates of compulsory psychiatric hospitalization are observed (6–9), which further underscores the importance of this issue. Despite its ethical and care-political relevance, up until now there has been a lack of preventive measures which, applied prior to a mental health crisis, are suited to reduce the number of compulsory admissions and to promote patients’ autonomy.

The significance of service users’ perceptions of coercion has only recently been recognized and incorporated into the broad discussion on shared decision-making models in psychiatry in various European countries. A number of studies have examined how common it is for patients in psychiatric care to experience the treatment they receive as coercive. Indeed, findings suggest that perceived coercion is strongly associated with a patient’s legal status at admission: according to a recent literature review, experienced coercion is more than eight times as likely in detained than in voluntarily admitted inpatients (10). It has also become evident, however, that “objective” legal status at admission and subjective feelings of coercion are not equivalent (11). Feeling coerced upon admission to a mental hospital may reflect a patient’s perceptions of having no choice about or control over entering the hospital, of negative pressures from other people, and of considering the hospital admission process as unfair (11–13). Hence, feelings of coercion are common whatsoever the legal status at admission (3). Whereas in the literature, prevalence rates for experienced coercion of 74% (95% CI, 63–82) on average have been found in legally detained patients (10, 14), 25% (95% CI, 20–31) of informally admitted inpatients also feel coerced into psychiatric care (10, 14), while a certain proportion of legally detained inpatients view their admission as largely voluntary (13, 15). This suggests that the formal legal status at admission is not the only etiological factor that accounts for variation in patients’ perceptions of coercion.

Among the factors that could account for differences in the patients’ views on the hospital admission process are low insight (15, 16), a hostile-dominant interpersonal style (2), being unmarried/not cohabiting (11, 16), as well as female sex (2, 11, 17), which have been found to correlate with (high) levels of perceived coercion. Psychotic disorder, too, repeatedly proved to be linked to high perceived coercion scores (15, 17, 18), whereas for other clinical variables (type of disorder, severity of symptoms, and chronicity) and sociodemographic factors (age and ethnicity), results are not conclusive.

Despite substantial research on coercive practices that has emerged in the past 20 years, our understanding of patients’ perception of coercion – and its opposite, patients’ perception of autonomy and empowerment – is still limited. Empowerment can be described as a process of gaining control over one’s life and building capacities to act on one’s own behalf (19, 20). The best way to define and measure empowerment, however, is still being debated and diverse definitions and conceptualizations of empowerment have been suggested (21, 22). In health promotion research and evaluation, the concept of empowerment has been related to processes not only at the individual level but also at the community level (e.g., the extent to which service users could influence organizational decisions). Rogers et al. (19, 23), who conducted large-scale surveys to investigate the level of empowerment among users of mental health services, identified five factors supposed to be constituent components of patient empowerment: self-esteem/self-efficacy, power, community activism, optimism/control over the future, and righteous anger. Empowerment is considered a key component of recovery (24, 25) and has been found to be related to quality of life (19).

A more in-depth understanding of empowerment and perceived coercion is crucial not only to understanding the factors that influence these views and behaviors but also in terms of their impact on treatment. Perceived coercion is linked to treatment satisfaction (26), and it is reasonable to expect that it may act as a barrier to accessing effective care in particular groups known to be dissatisfied with mental health services (15). Likewise, a study analyzing 1,543 admissions within a Finnish catchment area, of patients with severe mental disorder, has shown that a feeling of having been coerced into the admission is associated with less frequent use of medication and mental health centers’ services. The patients’ perception of involuntary admission also turned out to be more important in relation to satisfaction and outcome than their formal legal status (7). Given all this, perceived coercion should be regarded as an important outcome measure in service evaluation (15).

This article presents 12-month outcomes of an intervention program for patients with serious mental disorder who are at risk of involuntary placement. Through this intervention program, we aim to prevent compulsory readmission to psychiatric hospital treatment, to increase patients’ empowerment, and to reduce their level of perceived coercion. The service users’ perceptions therefore form an integral part of the outcome assessment of this trial. The study is implemented as a subproject within the framework of the Zurich Program for Sustainable Development of Mental Health Services (27). An analysis of compulsory readmissions to psychiatric hospital treatment during the first 12 months of this trial had suggested beneficial effects of the intervention program in terms of a lower number of compulsory readmissions among patients who had remained in the program for so long (28).

The present study addresses several questions: (A) to examine changes in the patients’ perspective, focusing on perceived coercion, empowerment, and self-reported mental health functioning. We hypothesized that the intervention alters patients’ perceived coercion so that they feel less coerced and experience greater empowerment. As to changes concerning symptomatic distress, we did a merely explorative analysis, since the intervention program did not primarily target reduction of clinical symptoms. (B) Furthermore, we seek to explore whether altered perceptions are linked to particular patient characteristics and (C) in which way the patients’ perceptions are related to the experience of compulsory rehospitalization during the 12-month period.

## Subjects and Methods

### Study Design

The design of the study and the intervention program are described elsewhere (8). In short, we undertook a randomized controlled trial to compare the intervention program with a treatment-as-usual (TAU) control condition. Patients with serious mental disorder who met the following criteria were included in the study: one or multiple compulsory admissions to psychiatry during the past 24 months, age 18–65, and a basic knowledge of the German language. Furthermore, patients had to be contactable by telephone post-release. Study participants were recruited from four psychiatric hospitals, all mandated to provide psychiatric care to adult patients in the Canton of Zurich, Switzerland, during an acute inpatient treatment episode. Patients diagnosed with an organic mental disorder (ICD-10: F0), mental retardation (F7), or a behavioral syndrome associated with physical factors (F5) were not included in the study. The study protocol was approved by the Ethical Review Board for Clinical Studies of Canton Zurich, Switzerland, and is registered with Current Controlled Trials ISRCTN63162737.

After having given informed consent to participate and randomization either to the intervention group or to a TAU group, all study participants completed a comprehensive baseline interview covering the patient’s perceptions, psychopathology, and service use. Baseline assessments included the patient’s subjective perspective on perceived coercion, empowerment, treatment satisfaction, quality of life, and various aspects of stigmatization. Follow-up assessments were scheduled 12 (t1) and 24 months (t2) after discharge from the hospital. In the follow-up interviews, almost all of these domains were addressed again.

To receive the most accurate and timely information possible as regards the participant’s rehospitalizations and further mental health care use (which will provide the basis for a cost analysis), we collected these data in regular monitoring phone calls. Participants assigned to the control group, which did not receive such monitoring, were called up every 3 months to briefly assess their service utilization over the past period in order to minimize recall errors.

### Intervention

The intervention program started with individualized psychoeducation focusing on behaviors prior to and during illness-related crises. Individual risk factors for relapse, personal and social resources, and use of mental health care services were assessed and compiled into an individual checklist. This checklist included familial, work, or financial problems as well as personal and social resources. With this information, a crisis card was compiled and handed out to the patient at discharge (29).

After discharge from the hospital, each participant in the intervention group was contacted by telephone every fourth week over a period of 24 months. At each contact, the patient’s present mental health status was assessed using the individual checklist. The monitoring covered the participant’s present living conditions, emotional state (signs of depression, withdrawal), suicidality, substance abuse, violence, as well as mental health service utilization (recent inpatient and outpatient psychiatric episodes, contacts to health care professionals, medication, etc.). If a relapse threatened to occur, a participant was reminded to activate his/her individual action plan (developed prior to discharge) and to contact his/her regular therapist or other mental health care services in order to prevent compulsory hospital readmission.

All parts of the program (psychoeducation and preventive monitoring) were carried out by a personal mental health care worker (graduated psychologist), who maintained the contact to the study participant over the course of the 24-month intervention. The monthly contacts during which the present situation is reviewed with the personal mental health care worker are designed to provide a dense individual pattern of the course of illness (to target early signs of a crisis) and the utilization of health care services.

This program primarily addresses the self-management skills of chronically mentally ill patients. It is supposed that patients who have experienced involuntary placement(s) in the past may have a strong interest to avoid further compulsory measures and therefore might benefit from such a program. The intervention program does not intend to replace the patients’ regular therapy; rather, it is considered a supplementary measure (supplementary to patients’ regular therapy) to give chronically mentally ill patients support to become more actively involved in their care. This individual long-term support offers opportunities to reflect on treatment-related behaviors (utilization of health care services; medication compliance) and to refer to ways of optimized service use in case of a relapse. By addressing the patients’ self-management skills, the program seeks to activate their potential for secondary prevention of relapses.

### Measures

To assess the patients’ perceptions of coercion during their admission to the psychiatric hospital, we used the *MacArthur Admission Experience Survey AES* [short form, 15 items; (11, 30)]. This questionnaire was also applied in a slightly modified version (adapted with reference to outpatient treatment) in the t1 assessment. All coercion questions were presented on a dichotomous true/false scale. We used these items to construct three subscales, as suggested in the original literature (11, 16), which characterize patients’ perceptions of perceived coercion (PercCo), of negative pressures (NegPr), and of process exclusion (ProcEx). For these subscales, high reliability and acceptable retest stability have been found (11). Higher scores on the AES subscales indicate higher levels of perceived coercion.

The *Empowerment–Scale* of Rogers et al. (19), which is widely used in mental health settings [e.g., see Ref. (24, 31–33)], was applied to measure empowerment and self-help involvement. This questionnaire consisting of 28 statements (four-point Likert scales ranging from “strongly agree” to “strongly disagree”) provides information on five domains: self-esteem, power, activism/autonomy, optimism, and righteous anger/control. The scale demonstrated a high degree of internal consistency. Empowerment is related to recovery, hope, and quality of life, but not to sociodemographic subject characteristics, except income (19, 23).

The *Outcome Questionnaire OQ-45* (34) was used to estimate changes in mental health functioning over time. This self-report questionnaire comprises 45 items to be rated by the patient on a five-point scale (0 “never” to 4 “almost always”). Three domains of functioning are assessed: symptomatic distress or subjective discomfort (SD), interpersonal relationships with intimate others (IR), and functioning in social roles such as work, homemaking, and leisure activities (SR). Studies on the psychometric properties of the OQ-45 suggest a high degree of retest reliability, internal consistency, and concurrent validity with scales such as the Symptom Checklist-90. Moreover, the three subscales and the total score have been found to be sensitive to the effects of interventions (35).

To arrive at subscale scores, we summed and averaged the items of the single AES, Empowerment, and OQ-45 domains. If a patient left an item blank, we used the average score for the remaining subscale items in place of the missing value.

Diagnostic and treatment-related data were taken from the patient files. The hospital physicians in charge at the participating study centers made the psychiatric diagnoses. Information on sociodemographic data and the patient’s history and utilization of mental health care services were gathered using the Client Socio-demographic and Service Receipt Inventory CSSRI-EU (36).

### Statistical Methods

For within-group and between-group comparisons of perceived coercion (AES scales), we applied non-parametric tests because the assumption of normal distribution is violated for these scores. Changes in individual ratings over time (t0 vs. t1 comparisons) therefore were analyzed by means of Wilcoxon signed-rank tests. For comparisons between treatment groups (intervention vs. TAU) or between participants with vs. those without compulsory rehospitalization, we applied Mann-Whitney *U* tests. As to empowerment and mental health functioning (Empowerment scale, OQ-45), we analyzed data by means of repeated-measures ANOVA. The significance level was fixed at 0.05.

In order to quantify the relative impact of potential explanatory variables on changes in outcome measures over time (perceived coercion, empowerment, and mental health functioning), we performed multiple regression analyses. In order to model changes, Δ-scores were calculated, all expressing the difference between t0-scores and t1-scores (thus resulting in negative scores if empowerment had increased up to t1). We fitted regression models only for those subscales which provided evidence of significant changes in Δ-scores. The baseline variables specified in Table 1 were considered explicative variables in the regression. All variables were entered in a single step. For an exploratory data analysis, we furthermore modeled the data, using forward stepwise variable selection. Criteria for variable selection in the stepwise procedures were a probability of F-to-enter of 0.05 and of F-to-remove of 0.15. All analyses were performed with SPSS 21.0.

**Table 1:** **Baseline sample characteristics**.

	Intervention	TAU
Sex, n (%)		
Female	71 (59.66)	62 (52.10)
Male	48 (40.34)	57 (47.90)
Age, years: M (SD)	41.53 (12.29)	43.36 (11.34)
Compulsory hospitalization due to		
Risk of self-harm	81 (68.07)	90 (75.63)
Risk of harm to others^a^	38 (31.93)	29 (24.37)
ICD-10 diagnosis		
Substance use disorder (F1)	24 (20.17)	23 (19.33)
Schizophrenia, mania, bipolar affective disorder (F2, F30-31)	41 (34.45)	52 (43.70)
Depressive, neurotic, stress related (F32-34, F4)	36 (30.25)	27 (22.69)
Personality disorder (F6)	18 (15.13)	17 (14.29)
Duration of illness, years: M (SD)	15.63 (12.55)	16.68 (12.50)
Psychiatric hospital admissions, n: M (SD)	8.52 (12.11)	9.32 (14.42)
Compulsory psychiatric admissions, n: M (SD)	3.84 (5.18)	4.79 (8.47)

*^a^Group with or without risk of self-harm*.

*TAU, treatment as usual; M, mean*.

*Intervention vs. TAU: no statistical significant group differences for these variables*.

## Results

### Sample Characteristics

Of the 756 psychiatric inpatients who were approached, 238 provided written informed consent and were randomized to the intervention group (*n* = 119) or a TAU group (*n* = 119). Table 1 shows the basic sample characteristics. Regarding its sociodemographic background, the sample is characterized by a low level of education (72.7% have only a basic education) and high rates of unemployment (68.9%) and participants living alone (53.4%).

Among the persons with psychotic disorder, there were 63 (26.5%) with schizophrenic disorder (ICD-10 F2) and 30 (12.6%) with mania or bipolar affective disorder (F30; F31). Of the study participants with personality disorder, 30 (12.6%) had been diagnosed with an “emotionally unstable personality disorder” (F60.3; in most cases Borderline personality disorder) or “mixed personality disorder” (F61). Across all diagnostic groups, psychiatric comorbidity was common: a substance use disorder in addition to another psychiatric main diagnosis, for example, was found in 80 study participants (33.6%). Corresponding to the severity of the disorders, most patients also suffered from major functional impairments.

At baseline assessment, the majority had already experienced a high number of previous hospitalizations (24.1% > 10 psychiatric hospitalizations). The index hospital admission was the first compulsory admission (lifetime) for 39.8% of the intervention group and for 37.3% of the TAU group; albeit almost every second participant (43.2% in the intervention group; 46.6% in the TAU group) had already experienced three or more compulsory admissions previously.

### Perceived Coercion, Empowerment, and Mental Health Functioning at Baseline and After 12 Months

Regarding the patients’ perceptions at baseline assessment (t0), as intended by randomization, there were no significant differences between the intervention group and the TAU group on any AES, empowerment, and mental health functioning subscale.

Of the baseline sample, 182 (76.5%) had remained in the program for a period of at least 12 months, whereas 56 were lost to t1 assessment. Of the 182 participants who attended the t1 interview, ≥95.6% completed the questionnaires (176, the AES; 175, the Empowerment scale; and 174, the OQ-45). The comparison of baseline scores did not reveal any significant difference between remainders who completed t1 assessments and dropouts.

Table 2 provides the t0 and t1 ratings of the 182 subjects who completed t1. After 12 months, the study participants reported lower levels of perceived coercion, NegPr, and ProcEx and a higher level of optimism. Moreover, answers suggest a lesser degree of distress due to symptoms, interpersonal relations, and social role functioning compared to the baseline assessment.

**Table 2:** **Perceived coercion, empowerment, and self-reported mental health functioning: effects of time, intervention group, and time × group interaction after 12 months**.

	Intervention	Intervention	TAU	TAU	Time	Group	Time ×group
	T_0_	T_1_	T_0_	T_1_			
	M (SD)	M (SD)	M (SD)	M (SD)	*p* value	*p* value	*p* value
**MacArthur admission experience survey AES (0–1)**
Perceived coercion	0.70 (0.41)	0.16 (0.25)	0.65 (0.39)	0.21 (0.30)	<0.001	0.503	0.102
Negative pressures	0.43 (0.33)	0.09 (0.17)	0.46 (0.36)	0.12 (0.24)	<0.001	0.652	0.904
Process exclusion	0.60 (0.36)	0.19 (0.27)	0.54 (0.38)	0.21 (0.30)	<0.001	0.409	0.216
**Empowerment (1–4)**
Self-esteem, self-efficacy	2.97 (0.62)	3.09 (0.59)	3.09 (0.56)	3.08 (0.62)	0.201	0.561	0.196
Power	2.50 (0.46)	2.48 (0.47)	2.50 (0.46)	2.48 (0.51)	0.634	0.769	0.762
Community activism, autonomy	3.37 (0.46)	3.46 (0.39)	3.49 (0.38)	3.45 (0.44)	0.396	0.352	0.143
Optimism, control over the future	2.94 (0.54)	3.12 (0.46)	3.01 (0.54)	3.13 (0.53)	0.001	0.714	0.595
Righteous anger	2.44 (0.63)	2.39 (0.56)	2.48 (0.60)	2.39 (0.62)	0.084	0.840	0.764
**OQ-45 (0–4)**
Symptom distress	1.57 (0.70)	1.42 (0.73)	1.51 (0.65)	1.29 (0.68)	<0.001	0.316	0.473
Interpersonal relations	1.42 (0.64)	1.23 (0.72)	1.50 (0.58)	1.33 (0.64)	<0.001	0.319	0.804
Social role	1.44 (0.65)	1.24 (0.63)	1.40 (0.63)	1.17 (0.63)	<0.001	0.511	0.773
Total score	1.48 (0.58)	1.30 (0.62)	1.47 (0.55)	1.26 (0.59)	<0.001	0.785	0.775

*p-values empowerment, OQ-45: repeated measurement variance analysis; p-values perceived coercion: Mann–Whitney U tests and Wilcoxon tests*.

Statistically, there was a significant change in patients’ perceptions in these domains (significant time effects), whereas we found no significant changes regarding empowerment, except for the subscale optimism. Improvements in these domains, however, were not confined to the intervention group, but also occurred at comparable levels in the TAU group (no significant group or interaction effect).

### Perceived Coercion, Empowerment, and Mental Health Functioning: Predictors of Altered Perceptions

Regression analyses, performed in order to examine which psychopathological and sociodemographic variables contribute to changes in patient perceptions, offered only a limited number of predictors. Variation in *perceived coercion* over 12 months was significantly associated with age (β = 0.207; *t* = 2.460; *p* = 0.015) and duration of illness (β = −0.251; *t* = −2.977; *p* = 0.003): the patients’ perceived coercion (PercCo) and perceived ProcEx (age: β = 0.251; *t* = 2.963; *p* = 0.004; duration of illness: β = −0.268; *t* = −3.143; *p* = 0.002) declined from t0 to t1 assessment with increasing age and a shorter duration of the mental disorder (Table 3). Likewise, using stepwise regression, of all candidate variables considered, only duration of illness (β = −0.252; *t* = −3.122; *p* = 0.002) and age (β = 0.220; *t* = 2.730; *p* = 0.007) were found to predict change over time with respect to perceived coercion.

**Table 3:** **Perceived coercion and empowerment: predictors of changes (regression analysis, standardized regression coefficients, and 95% confidence intervals)**.

	AES			Empowerment
	Δ PercCo	Δ NegPr	Δ ProcEx	Δ Optimism
	Beta (95% CI)	Beta (95% CI)	Beta (95% CI)	Beta (95% CI)
Treatment group, intervention	0.097 (−0.051; 0.231)	0.021 (−0.101; 0.132)	0.074 (−0.067; 0.192)	−0.008 (−0.189; 0.169)
Sex, male	0.008 (−0.135; 0.150)	−0.104 (−0.195; 0.040)	−0.093 (−0.210; 0.053)	**0.170 (0.019; 0.381)**
Age (years)	**0.207 (0.002; 0.015)**	0.016 (−0.005; 0.006)	**0.251 (0.003; 0.015)**	−0.118 (−0.014; 0.002)
Compulsory hospitalization due to risk of harm to others	−0.041 (−0.205; 0.123)	0.079 (−0.071; 0.199)	0.118 (−0.043; 0.261)	−0.046 (−0.269; 0.150)
ICD-10 diagnosis				
Schizophrenia, mania, bipolar disorder (F2, F30-31)	−0.044 (−0.204; 0.122)	0.090 (−0.067; 0.201)	0.015 (−0.138; 0.163)	**0.185 (0.012; 0.425)**
Personality disorder (F6)	−0.026 (−0.275; 0.202)	−0.064 (−0.268; 0.124)	0.012 (−0.203; 0.233)	−0.011 (−0.321; 0.280)
Duration of illness (years)	**−0.251 (−0.015; −0.003)**	−0.042 (−0.006; 0.004)	**−0.268 (−0.015; −0.033)**	0.059 (−0.005; 0.011)
Compulsory psychiatric admissions (n; lifetime)	0.056 (−0.006; 0.012)	0.120 (−0.002; 0.013)	0.021 (−0.007; 0.009)	0.037 (−0.009; 0.014)

*AES, MacArthur Admission Experience Survey; PercCo, perceived coercion; NegPr, negative pressures; ProcEx, process exclusion*.

*Statistical significant coefficients are written in bold*.

Regarding outcomes of the Empowerment scale, *optimism* (the only domain of the Empowerment scale that markedly improved over time) was significantly related to “sex” (β = 0.170; *t* = 2.178; *p* =0.031) and “psychiatric diagnosis” (β = 0.185; *t* = 2.085; *p* = 0.039). Results suggest that in male patients, compared to female, and in patients with psychotic disorder, compared to other diagnoses, changes on the optimism scale were heading for less increase in optimism up to t1 assessment. Accordingly, being diagnosed with a psychotic disorder (β = 0.193; *t* = 2.580; *p* = 0.011) and of male sex (β = 0.176; *t* = 2.362; *p* = 0.019) were the only variables selected in stepwise multiple regression.

Changes in the patients’ perceptions of their *mental health functioning* were associated with only one variable, namely the reason for their compulsory hospitalization (risk of harm to others vs. risk of self-harm). In the simultaneous analysis, compulsory hospitalization due to risk of violent behavior was significantly associated with a less marked decline in social role difficulties (SR; β = −0.183; *t* = −2.213; *p* = 0.028), holding constant scores on all other variables in the model (Table 4). This was also the only predictor selected in the stepwise procedure: being compulsorily admitted “due to risk of harm to others” was associated with less decrease in symptom distress (SD), SR, and OQ-45 total score over time, compared to being compulsorily admitted “due to risk of self-harm” (SD: β = −0.195; *t* = −2.564; *p* = 0.011; SR: β = −0.239; *t* = −3.180; *p* = 0.002; Total: β = −0.196; *t* = −2.580; *p* = 0.011).

**Table 4:** **Self-reported mental health functioning: predictors of changes (regression analysis, standardized regression coefficients, and 95% confidence intervals)**.

	OQ-45			
	Δ SD	Δ IR	Δ SR	Δ Total
	Beta (95% CI)	Beta (95% CI)	Beta (95% CI)	Beta (95% CI)
Treatment group, intervention	−0.066 (−0.249; 0.098)	0.026 (−0.166; 0.231)	−0.023 (−0.231; 0.170)	−0.024 (−0.183; 0.135)
Sex, male	−0.138 (−0.334; 0.019)	−0.050 (−0.264; 0.139)	−0.118 (−0.359; 0.047)	−0.121 (−0.287; 0.036)
Age (years)	0.086 (−0.004; 0.012)	0.069 (−0.006; 0.013)	0.043 (−0.007; 0.012)	0.078 (−0.004; 0.011)
Compulsory hospitalization due to risk of harm to others	−0.118 (−0.352; 0.057)	−0.066 (−0.325; 0.143)	**−0.183 (−0.499; −0.028)**	−0.148 (−0.354; 0.020)
ICD-10 diagnosis				
Schizophrenia, mania, bipolar disorder (F2, F30-31)	−0.139 (−0.361; 0.043)	0.045 (−0.177; 0.288)	−0.133 (−0.409; 0.056)	−0.089 (−0.278; 0.092)
Personality disorder (F6)	−0.070 (−0.412; 0.171)	0.014 (−0.308; 0.360)	−0.067 (−0.469; 0.203)	−0.049 (−0.343; 0.191)
Duration of illness (years)	−0.037 (−0.009; 0.006)	0.019 (−0.008; 0.010)	0.064 (−0.005; 0.012)	0.022 (−0.006; 0.008)
Compulsory psychiatric admissions (n; lifetime)	−0.011 (−0.012; 0.010)	0.013 (−0.012; 0.014)	0.030 (−0.010; 0.015)	0.014 (−0.009; 0.011)

*OQ-45, Outcome Questionnaire; SD, symptom distress; IR, interpersonal relations; SR, social role*.

*Statistical significant coefficients are written in bold*.

### Comparison of Study Participants with and without Compulsory Rehospitalizations

During the 12 months up to t1 assessment, 18 patients (22.5%) in the intervention group and 36 (35.3%) in the TAU group had one or more further compulsory admissions after having been discharged from the hospital (28). In order to explore the extent to which the patients’ perceptions of coercion coincide with such an experience, we compared study participants with compulsory rehospitalization(s) to those without.

In the intervention group, t1-levels of perceived coercion, NegPr, and ProcEx tended to be higher in study participants with compulsory rehospitalization (mean PercCo, 0.29; NegpPr, 0.15; ProcEx, 0.30) than in participants in the intervention group without compulsory rehospitalization (0.13; 0.08; 0.16), but results do not reach statistical significance.

In the TAU group, too, study participants with compulsory rehospitalization scored higher on all AES subscales (PercCo, 0.28; NegpPr, 0.22; ProcEx, 0.29) than participants in the TAU group without compulsory rehospitalization (0.18; 0.08; 0.16), and these group differences are statistically significant (PercCo *U* = −2.202, *p* = 0.028; NegpPr *U* = −3.318, *p* = 0.001; ProcEx *U* = −2.002, *p* = 0.045).

As regards other t1-measures (empowerment, mental health functioning), there was virtually no notable difference between study participants with and without compulsory rehospitalizations.

## Discussion

To determine the effects of an intervention program that addresses the reduction of compulsory hospitalization in people with severe mental disorder, we analyzed changes in the patients’ perceived coercion, empowerment, and self-reported mental health functioning after 12 months. Whereas we found lower levels of patients’ perceived coercion (AES subscales perceived coercion, NegPr, ProcEx), there were no significant changes in empowerment domains, except optimism. Lower levels of perceived coercion and SD, however, were seen not only in the intervention group but virtually to the same degree also in the TAU group.

What are possible explanations for these findings? Considering that the majority of the sample was suffering from long-lasting severe mental health problems, it is not to be assumed that feeling less coerced and more optimistic would have occurred anyway in a matter of time. Rather, it appears that patients who agreed to participate in such a long-term project, which could be expected to make (temporally as well as mentally) great demands on them, were especially motivated to undertake substantial efforts to avoid compulsory rehospitalization.

This initial attitude apparently persisted over the course of the study, irrespective of whether a person had been assigned to the intervention group or to the control group. Participants in the control group also had a strong interest in avoiding compulsory rehospitalization. Our follow-up interviews (the purpose of which was to surveil patients’ mental health care use prospectively) might have reinforced their feeling themselves part of this prevention program and their feelings of being helped: grappling regularly with this topic therefore might have activated coping strategies and promoted a spill-over effect in terms of a shift of views toward having more control over the future and feeling less coerced, also in control group participants. Such effects are well known in clinical psychology, e.g., from family therapy (37), the prevention of stress-related diseases (38), or in terms of motivational–behavioral interfaces (39) in health psychology, where positive (intended or unintended) spill-over effects have been observed.

Another issue to consider when interpreting TAU effects is that, unlike clinical trials in medicine, health services research is faced with more methodological complexities. Some of these have to do with the fact that data, such as outcome measures of perceived coercion or empowerment, are “soft.” Besides, boundary conditions that might have a bearing on outcomes in the course of such a long-term intervention can hardly be controlled. Unlike in RCTs, in which an investigator gives the research subjects a particular medicine or does not, the factors determining progress in a complex “case management” program are less clear-cut. A broad range of treatment options and mental health care services are available to people with mental problems in the Canton of Zurich. For example, 11 psychiatric institutions provided community mental health services (40) and a total of 762 psychiatrists, 587 of them in outpatient care (41), served a population of circa 1.42 million people in 2013. Thus, “treatment as usual” alone can draw on ample resources in this mental health care system, with the result that positive treatment effects are to be expected also in TAU patients.

Perceived coercion so far has been addressed (as a secondary outcome measure) primarily in the context of clinical trials that examined the effectiveness of Joint Crisis Plans. Results of these studies cannot, of course, be directly compared. It might be worth mentioning though that these studies likewise did not suggest significant treatment effects in patients with borderline personality disorder (42), or in people with psychosis, respectively (43), compared to control groups.

In all empowerment domains, except optimism, we failed to detect significant changes over time. This was contrary to our expectations, particularly because our intervention program explicitly addressed the participants’ self-management skills, encouraging their active involvement in their treatment. A possible explanation might relate to the Empowerment scale applied. It is obvious that the meaning of empowerment may differ across different populations of people with mental health problems and in different health care contexts (24). The *Empowerment–Scale* of Rogers et al. (19) was developed from the perspective of consumer activists and consumer-operated programs (23), and this special connotation might have rendered it not the most appropriate instrument for measuring the changes intended here. Moreover, the items can be read as if a personal attitude were being considered rather than a personal appraisal of one’s present status. Besides these methodical aspects, there is evidence from various studies that high levels of psychiatric symptoms may serve as barriers to empowerment (23, 32, 44). “Individuals may experience difficulty achieving high levels of empowerment unless symptoms of the psychiatric illness are addressed,” as pointed out by Strack and Schulenberg (32). Considering that the current sample included people with severe mental health problems and major impairment of social functioning, which were still prevalent after 12 months, the lack of intervention effects in terms of an increase in empowerment may be ascribed to these factors.

A further reason might relate to the nature of the intervention provided. This psychoeducation-based approach can be considered a form of intensive case management by telephone, targeting the early recognition of relapse and prevention of compulsory rehospitalization. The intervention program, however, did not include psychotherapy, and it was not intended to take the place of regular psychiatric treatment. It is therefore likely that a more intensive form of treatment would have been required in order to enhance self-esteem, self-efficacy, and perceived empowerment in these patients. Even with the most promising therapeutic approaches, as, e.g., cognitive behavioral therapy or positive psychotherapy, however, only small and statistically non-significant effects were achieved as regards the patients’ empowerment (45, 46). Taken together, these findings suggest that whatever treatment method was used, demonstrable improvement in “objective” outcomes was not reflected in the patients’ perception of empowerment. This lack of correspondence leaves room for different interpretations: it might reflect the ambiguity of the concept of empowerment itself, or, alternatively, subjective views of empowerment might only loosely be linked to behavioral or functional changes.

There are several limitations to this study. First, it has to be stressed that the sample included in this study is not representative of psychiatry patients in total considering their functional impairment, severity of symptoms, and the wide range of comorbid conditions. Moreover, participation in this study was voluntary and, of all inpatients approached, only about one in three agreed to participate. It is to be assumed that this sample consists of highly motivated individuals. Therefore, generalizability of these findings is limited. Second, this is an analysis of the results over the first 12 months (t1); shifts in the patients’ evaluations of coercion and empowerment therefore may still occur. However, considering that a significantly lower number of compulsory readmissions per patient registered after 12 months of participation in the monitoring program (28) did not go in parallel with changes in the patients’ subjective perspective (perceived coercion and empowerment) so far, it is unlikely that convergence between subjective and objective outcomes will evolve before t2. Moreover, the lack of treatment effects on these outcome measures is in line with results of earlier research, as outlined above. A further limitation relates to the analysis, which reflects the outcomes only of those study participants who have remained in the program so far. Like all as-treated analyses, it ignores bias that might be associated with dropout. There is no indication though that study remainders who completed t1 questionnaires differ from dropouts at t1 on any baseline ratings or clinical variables. It is therefore unlikely that the study participants’ initial perceived coercion and empowerment might explain early dropout from the trial. Finally, a general limitation of this study can be seen in the fact that this intervention exclusively targets individual risk factors that are subject to the patient’s self-management while service system aspects (referral, crisis intervention procedures, etc.) that might too have a share in involuntary placement and perceived coercion are not addressed.

The strengths of this study include its prospective design, the use of objective and subjective outcome criteria in order to evaluate intervention effects, and its implementation under real community mental health conditions. The participation rate and the rate of 76% that remained in the study over a period of 12 months suggests that the program appeals to patients with a broad spectrum of severe psychiatric conditions and at risk of compulsory hospitalization.

Considering that this study is the first to evaluate this new intervention, data are on a provisional basis unless findings will be confirmed in a subsequent replication study. The present analysis focusing on the subjective patient evaluations, however, does not suggest effectiveness of the preventive monitoring program at this point in terms of an increase in patients’ empowerment or a reduction in their perceived coercion (beyond unspecific effects of participation in this trial). Further research therefore is required to find effective methods to enhance self-esteem and empowerment and to reduce perceived coercion in individuals with serious mental health problems and a history of voluntary and involuntary hospitalizations. For these high-risk patients whose particular needs are not adequately met by general psychiatry today, future research will need to develop and to test new types of preventive strategies if shared decision making as a central feature of patient-centered care, that is increasingly advocated as a guide for modern mental health care, is to be taken seriously.

## Author Contributions

BL and WR designed the study. TD, MB, SL, and CB coordinated the intervention and the data collection. BL performed the statistical analyses and drafted the manuscript. TD, MB, SL, CB, and WR interpreted the results and revised the manuscript for important intellectual content. All authors read and approved the final manuscript.

## Conflict of Interest Statement

The authors declare that the research was conducted in the absence of any commercial or financial relationships that could be construed as a potential conflict of interest.

## Abbreviations

AES, MacArthur Admission Experience Survey; CSSRI-EU, Client Socio-demographic and Service Receipt Inventory; IR, interpersonal relationships with intimate others (OQ-45); NegPr, negative pressures (AES); OQ-45, Outcome Questionnaire; PercCo, perceived coercion (AES); ProcEx, process exclusion (AES); SD, subjective discomfort (OQ-45); SR, functioning in social roles (OQ-45); ZInEP, Zurich Program for Sustainable Development of Mental Health Services.
